# (2*E*)-2-[2-(4-Chloro­phen­yl)hydrazin-1-yl­idene]-4,4,4-trifluoro-3-oxobutanal

**DOI:** 10.1107/S1600536810021835

**Published:** 2010-06-16

**Authors:** Yan-Ping Huo, Li-Hua Zhou

**Affiliations:** a Faculty of Chemical Engineering and Light Industry, Guangdong University of Technology, Guangzhou 510006, People’s Republic of China

## Abstract

The title compound, C_10_H_6_ClF_3_N_2_O_2_, was synthesized by coupling 4-dimethyl­amino-1,1,1-trifluoro­but-3-en-2-one with 4-chloro­benzene­diazo­nium chloride. It crystallizes with two mol­ecules in the asymmetric unit, which form two similar centrosymmetric dimers *via* hydrogen bonds. Extensive electron delocalization and intra­molecular N—H⋯O hydrogen bonds are responsible for a planar conformation of the mol­ecules (maximum deviations = 0.010 and −0.015 Å for the two molecules). In addition to hydrogen bonds, π–π stacking inter­actions with centroid–centroid distances of 3.604 (2) and 3.583 (2) Å contribute to the stability of the crystal structure.

## Related literature

For the crystal structure of the isostructural iodo derivative, see: Jiang & Zhu (2008 ▶).
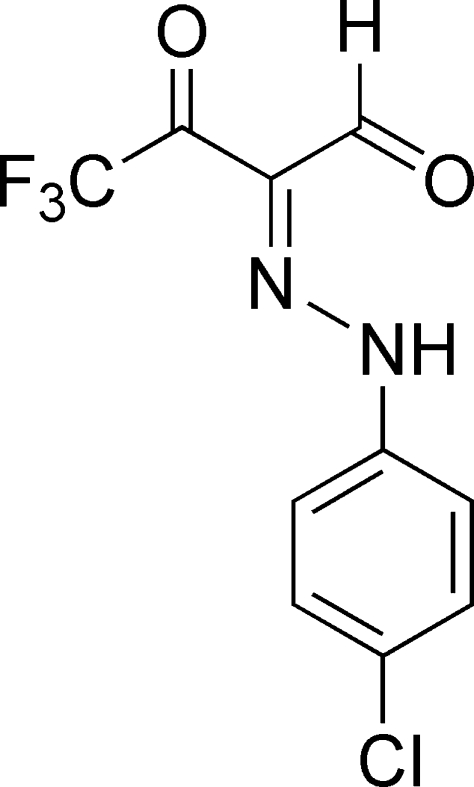


## Experimental

### 

#### Crystal data

C_10_H_6_ClF_3_N_2_O_2_
*M* *_r_* = 278.62
Triclinic, 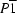

*a* = 7.6440 (4) Å
*b* = 7.7139 (4) Å
*c* = 19.4221 (10) Å
α = 86.134 (1)°
β = 81.706 (1)°
γ = 88.999 (1)°
*V* = 1130.63 (10) Å^3^
*Z* = 4
Mo *K*α radiation
μ = 0.37 mm^−1^
*T* = 173 K
0.44 × 0.38 × 0.35 mm

#### Data collection

Bruker SMART 1000 CCD diffractometer
Absorption correction: multi-scan (*SADABS*; Bruker, 2001 ▶) *T*_min_ = 0.853, *T*_max_ = 0.880
8820 measured reflections
4387 independent reflections
3577 reflections with *I* > 2σ(*I*)
*R*_int_ = 0.017

#### Refinement

*R*[*F* ^2^ > 2σ(*F* ^2^)] = 0.033
*wR*(*F* ^2^) = 0.111
*S* = 1.05
4387 reflections
325 parameters
H-atom parameters constrained
Δρ_max_ = 0.29 e Å^−3^
Δρ_min_ = −0.23 e Å^−3^

### 

Data collection: *SMART* (Bruker, 2001 ▶); cell refinement: *SAINT-Plus* (Bruker, 2003 ▶); data reduction: *SAINT-Plus*; program(s) used to solve structure: *SHELXS97* (Sheldrick, 2008 ▶); program(s) used to refine structure: *SHELXL97* (Sheldrick, 2008 ▶); molecular graphics: *ORTEP-3 for Windows* (Farrugia, 1997 ▶); software used to prepare material for publication: *SHELXL97*.

## Supplementary Material

Crystal structure: contains datablocks I, global. DOI: 10.1107/S1600536810021835/gk2268sup1.cif

Structure factors: contains datablocks I. DOI: 10.1107/S1600536810021835/gk2268Isup2.hkl

Additional supplementary materials:  crystallographic information; 3D view; checkCIF report

## Figures and Tables

**Table 1:** Hydrogen-bond geometry (Å, °)

*D*—H⋯*A*	*D*—H	H⋯*A*	*D*⋯*A*	*D*—H⋯*A*
N11—H11⋯O8	0.88	2.01	2.6746 (18)	131
N29—H29⋯O26	0.88	2.03	2.679 (2)	130
N29—H29⋯O26^i^	0.88	2.42	3.2159 (19)	150
C27—H27⋯O6^ii^	0.95	2.59	3.491 (2)	158
C36—H36⋯O26^i^	0.95	2.52	3.323 (3)	143

Symmetry codes: (i) 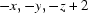; (ii) 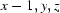.

**Fig. 1.**
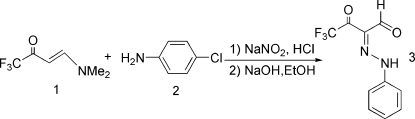
The synthesis of (E)-2-(2-(4-Chlophenyl)hydrazono)-4,4,4-trifluoro-3-oxobutanal

**Fig. 2.**
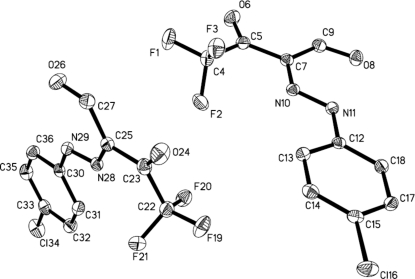
View of the asymmetric unit in the title compound.

## supplementary crystallographic information

### Comment

Herein, we report the crystal structure of (2*E*)-2-[2-(4-chlorophenyl)]hydrazinylidene]-4,4,4-trifluoro-3-oxobutanal, which was prepared *via* a reaction of 4-(dimethylamino)-1,1,1-trifluorobut-3-en-2-one with diazonium salt according to the procedure reported by Zhu *et al. *(2008). The title compound, 3, has been characterized by ESI-MS, NMR, FTIR spectroscopy and elemental analysis. Here we report the crystal structure of 3. It crystallizes with two almost identical molecules in the asymmetric unit. The molecule is almost planar except for the –CF_3_ group F atoms. There are some supramolecular interactions in the compound 3. The intramolecular N—H···O hydrogen bonds are N11—H11···O8 and N29—H29···O26 (Table 1) together with strong π-π stacking interactions [centroid-to-centroid distance = 3.604 (2) Å; 3.583 (2) Å] that contribute to the stability of the structure.

### Experimental

The title compound was prepared *via* the reaction of 4-(dimethylamino)-1,1,1-trifluorobut-3-en-2-one with diazonium salt according to the procedure reported by Zhu *et al. *(2008). A solution of the *p*-chloroaniline 2 (1.28 g, 10 mmol) in a solution of 3 *M* HCl (5 ml) was diazotized at 0 °C by slow addition of a solution of NaNO_2_ (0.7 g, 10 mmol) in 5 ml H_2_O. The solution of aniline diazonium salt was added dropwise to a mixture of compound 1 (see scheme) (1.67 g, 10 mmol) with NaOH (1.6 g, 40 mmol) and ethanol (50 ml) in ice-salt bath. The reaction mixture was stirred for 1 h at the same temperature, then TLC analysis showed that the reaction had finished. The resulting precipitate was filtered off. Purification by column chromatography on silica gel (hexane:AcOEt = 30:1) gave red solid 3 in 75% yield. mp 418-420 K. ^1^H NMR (CDCl_3_, 300 MHz) δ14.87 (1*H*, s, NH), 10.03 (1*H*, s, CHO), 7.45 (4*H*, s, Ph), ^19^F NMR (CDCl_3_): -71.50 (3 F, s, CF_3_). IR (KBr, cm^-1^): 2924, 1699, 1526, 1308, 1187, 1157, 897; ESI-MS *m*/*z*: 279.9 ([*M*+H]^+^); Elemental analysis: found C: 43.13, H: 2.29, N: 10.07; calculated for (C_10_H_6_ClF_3_N_2_O_2_) C: 43.11 H: 2.17 N: 10.05 (%). 20 mg of compound 3 was dissolved in 10 ml (EtOAc:pPetroleum ether = 1:8) and the solution was kept at room temperature for 6 d, natural evaporation gave red single crystals of compound 3 suitable for X-ray analysis.

### Refinement

All H atoms were positioned geometrically and refined using a riding model, with all C—H = 0.95 Å, N—H = 0.88 Å and with *U*_ĩso_(H) = 1.2*U*_eq_(C), *U*_ĩso_(H) = 1.2*U*_eq_(N).

### Crystal data

**Table:**

C_10_H_6_ClF_3_N_2_O_2_	*Z* = 4
*M**_r_* = 278.62	*F*(000) = 560
Triclinic, *P*1	*D*_x_ = 1.637 Mg m^−^^3^
Hall symbol: -P 1	Mo *K*α radiation, λ = 0.71073 Å
*a* = 7.6440 (4) Å	Cell parameters from 8820 reflections
*b* = 7.7139 (4) Å	θ = 1.1–26.0°
*c* = 19.4221 (10) Å	µ = 0.37 mm^−^^1^
α = 86.134 (1)°	*T* = 173 K
β = 81.706 (1)°	Block, yellow
γ = 88.999 (1)°	0.44 × 0.38 × 0.35 mm
*V* = 1130.63 (10) Å^3^	

### Data collection

**Table:**

Bruker SMART 1000 CCD diffractometer	4387 independent reflections
Radiation source: fine-focus sealed tube	3577 reflections with *I* > 2σ(*I*)
graphite	*R*_int_ = 0.017
ω scans	θ_max_ = 26.0°, θ_min_ = 1.1°
Absorption correction: multi-scan (*SADABS*; Bruker, 2001)	*h* = −9→9
*T*_min_ = 0.853, *T*_max_ = 0.880	*k* = −9→9
8820 measured reflections	*l* = −23→23

### Refinement

**Table:**

Refinement on *F*^2^	Primary atom site location: structure-invariant direct methods
Least-squares matrix: full	Secondary atom site location: difference Fourier map
*R*[*F*^2^ > 2σ(*F*^2^)] = 0.033	Hydrogen site location: inferred from neighbouring sites
*wR*(*F*^2^) = 0.111	H-atom parameters constrained
*S* = 1.05	*w* = 1/[σ^2^(*F*_o_^2^) + (0.0688*P*)^2^ + 0.2643*P*] where *P* = (*F*_o_^2^ + 2*F*_c_^2^)/3
4387 reflections	(Δ/σ)_max_ = 0.001
325 parameters	Δρ_max_ = 0.29 e Å^−^^3^
0 restraints	Δρ_min_ = −0.23 e Å^−^^3^

### Special details

**Table:**

Geometry. All esds (except the esd in the dihedral angle between two l.s. planes) are estimated using the full covariance matrix. The cell esds are taken into account individually in the estimation of esds in distances, angles and torsion angles; correlations between esds in cell parameters are only used when they are defined by crystal symmetry. An approximate (isotropic) treatment of cell esds is used for estimating esds involving l.s. planes.
Refinement. Refinement of F^2^ against ALL reflections. The weighted R-factor wR and goodness of fit S are based on F^2^, conventional R-factors R are based on F, with F set to zero for negative F^2^. The threshold expression of F^2^ > 2sigma(F^2^) is used only for calculating R-factors(gt) etc. and is not relevant to the choice of reflections for refinement. R-factors based on F^2^ are statistically about twice as large as those based on F, and R- factors based on ALL data will be even larger.

### Fractional atomic coordinates and isotropic or equivalent isotropic displacement parameters (Å^2^)

**Table:**

	*x*	*y*	*z*	*U*_iso_*/*U*_eq_	
F1	0.41928 (17)	−0.14617 (18)	0.81983 (6)	0.0571 (4)	
F2	0.43024 (15)	0.08932 (15)	0.75482 (6)	0.0461 (3)	
F3	0.36230 (15)	−0.15031 (16)	0.71485 (6)	0.0464 (3)	
C4	0.4666 (3)	−0.0800 (3)	0.75480 (10)	0.0373 (4)	
C5	0.6660 (2)	−0.1153 (2)	0.73104 (9)	0.0312 (4)	
O6	0.7481 (2)	−0.19276 (19)	0.77259 (7)	0.0478 (4)	
C7	0.7407 (2)	−0.0528 (2)	0.66091 (9)	0.0271 (4)	
O8	0.99555 (16)	−0.04137 (17)	0.57727 (7)	0.0362 (3)	
C9	0.9266 (2)	−0.0873 (2)	0.63594 (10)	0.0315 (4)	
H9	0.9963	−0.1478	0.6666	0.038*	
N10	0.62895 (18)	0.03374 (17)	0.62508 (7)	0.0258 (3)	
N11	0.67864 (18)	0.09586 (17)	0.56194 (7)	0.0255 (3)	
H11	0.7879	0.0804	0.5417	0.031*	
C12	0.5552 (2)	0.18901 (19)	0.52582 (8)	0.0244 (3)	
C13	0.3785 (2)	0.2002 (2)	0.55450 (9)	0.0287 (4)	
H13	0.3384	0.1459	0.5992	0.034*	
C14	0.2614 (2)	0.2914 (2)	0.51727 (10)	0.0316 (4)	
H14	0.1398	0.2989	0.5359	0.038*	
C15	0.3231 (2)	0.3710 (2)	0.45304 (9)	0.0290 (4)	
Cl16	0.17686 (7)	0.48727 (6)	0.40584 (3)	0.04196 (15)	
C17	0.4984 (2)	0.3604 (2)	0.42417 (9)	0.0296 (4)	
H17	0.5382	0.4158	0.3796	0.036*	
C18	0.6156 (2)	0.2678 (2)	0.46094 (9)	0.0287 (4)	
H18	0.7367	0.2585	0.4417	0.034*	
F19	0.14628 (17)	0.47780 (18)	0.67586 (6)	0.0541 (3)	
F20	0.32094 (14)	0.45871 (15)	0.75272 (6)	0.0422 (3)	
F21	0.06433 (17)	0.57720 (15)	0.77605 (7)	0.0525 (3)	
C22	0.1519 (2)	0.4478 (3)	0.74380 (10)	0.0368 (4)	
C23	0.0728 (2)	0.2668 (2)	0.76964 (10)	0.0344 (4)	
O24	0.0173 (2)	0.1827 (2)	0.72714 (8)	0.0522 (4)	
C25	0.0720 (2)	0.2110 (2)	0.84266 (9)	0.0302 (4)	
O26	0.0099 (2)	−0.01845 (17)	0.92755 (7)	0.0438 (3)	
C27	0.0109 (2)	0.0371 (2)	0.86723 (10)	0.0367 (4)	
H27	−0.0294	−0.0355	0.8353	0.044*	
N28	0.13267 (18)	0.32559 (19)	0.88092 (7)	0.0287 (3)	
N29	0.13874 (19)	0.29289 (19)	0.94648 (7)	0.0295 (3)	
H29	0.1023	0.1924	0.9673	0.035*	
C30	0.2050 (2)	0.4213 (2)	0.98458 (9)	0.0287 (4)	
C31	0.2464 (2)	0.5856 (2)	0.95348 (10)	0.0346 (4)	
H31	0.2305	0.6132	0.9064	0.042*	
C32	0.3108 (3)	0.7084 (3)	0.99149 (11)	0.0391 (4)	
H32	0.3400	0.8212	0.9707	0.047*	
C33	0.3327 (2)	0.6661 (3)	1.06009 (10)	0.0367 (4)	
Cl34	0.41607 (7)	0.82172 (8)	1.10737 (3)	0.05462 (18)	
C35	0.2909 (3)	0.5042 (3)	1.09141 (10)	0.0397 (4)	
H35	0.3058	0.4773	1.1387	0.048*	
C36	0.2265 (2)	0.3804 (3)	1.05305 (10)	0.0355 (4)	
H36	0.1974	0.2677	1.0739	0.043*	

### Atomic displacement parameters (Å^2^)

**Table:**

	*U*^11^	*U*^22^	*U*^33^	*U*^12^	*U*^13^	*U*^23^
F1	0.0558 (8)	0.0763 (9)	0.0332 (7)	−0.0031 (7)	0.0083 (6)	0.0094 (6)
F2	0.0472 (7)	0.0443 (7)	0.0457 (7)	0.0121 (5)	−0.0005 (5)	−0.0122 (5)
F3	0.0399 (6)	0.0555 (7)	0.0441 (7)	−0.0124 (5)	−0.0044 (5)	−0.0055 (5)
C4	0.0405 (11)	0.0413 (10)	0.0281 (9)	−0.0002 (8)	0.0008 (8)	−0.0012 (8)
C5	0.0361 (9)	0.0271 (8)	0.0306 (9)	0.0005 (7)	−0.0056 (8)	−0.0019 (7)
O6	0.0513 (9)	0.0526 (9)	0.0378 (8)	0.0088 (7)	−0.0084 (7)	0.0115 (7)
C7	0.0293 (8)	0.0254 (8)	0.0274 (9)	0.0005 (7)	−0.0058 (7)	−0.0031 (6)
O8	0.0294 (7)	0.0437 (7)	0.0344 (7)	0.0009 (6)	−0.0017 (5)	−0.0011 (6)
C9	0.0299 (9)	0.0311 (9)	0.0344 (10)	0.0017 (7)	−0.0068 (8)	−0.0031 (7)
N10	0.0293 (7)	0.0225 (7)	0.0262 (7)	−0.0011 (6)	−0.0056 (6)	−0.0028 (5)
N11	0.0238 (7)	0.0257 (7)	0.0267 (7)	−0.0001 (5)	−0.0031 (6)	−0.0007 (6)
C12	0.0289 (8)	0.0188 (7)	0.0266 (8)	0.0002 (6)	−0.0071 (7)	−0.0035 (6)
C13	0.0307 (9)	0.0256 (8)	0.0290 (9)	−0.0008 (7)	−0.0018 (7)	−0.0008 (7)
C14	0.0269 (9)	0.0285 (9)	0.0398 (10)	0.0013 (7)	−0.0044 (7)	−0.0050 (7)
C15	0.0344 (9)	0.0235 (8)	0.0318 (9)	0.0047 (7)	−0.0129 (7)	−0.0052 (7)
Cl16	0.0452 (3)	0.0363 (3)	0.0482 (3)	0.0099 (2)	−0.0215 (2)	−0.0017 (2)
C17	0.0369 (9)	0.0266 (8)	0.0255 (9)	0.0000 (7)	−0.0056 (7)	−0.0012 (7)
C18	0.0282 (8)	0.0281 (8)	0.0294 (9)	0.0005 (7)	−0.0020 (7)	−0.0028 (7)
F19	0.0567 (8)	0.0720 (9)	0.0322 (6)	−0.0073 (6)	−0.0087 (5)	0.0149 (6)
F20	0.0355 (6)	0.0476 (6)	0.0424 (6)	−0.0043 (5)	−0.0058 (5)	0.0071 (5)
F21	0.0563 (8)	0.0395 (6)	0.0557 (8)	0.0145 (6)	0.0049 (6)	0.0082 (6)
C22	0.0339 (10)	0.0426 (11)	0.0326 (10)	0.0036 (8)	−0.0030 (8)	0.0024 (8)
C23	0.0291 (9)	0.0417 (10)	0.0321 (10)	0.0027 (8)	−0.0039 (7)	−0.0026 (8)
O24	0.0641 (10)	0.0588 (9)	0.0367 (8)	−0.0119 (8)	−0.0154 (7)	−0.0049 (7)
C25	0.0281 (9)	0.0313 (9)	0.0302 (9)	0.0021 (7)	−0.0015 (7)	−0.0020 (7)
O26	0.0559 (9)	0.0368 (7)	0.0379 (8)	−0.0070 (6)	−0.0066 (6)	0.0050 (6)
C27	0.0389 (10)	0.0337 (10)	0.0374 (11)	−0.0014 (8)	−0.0037 (8)	−0.0041 (8)
N28	0.0245 (7)	0.0328 (8)	0.0277 (8)	0.0039 (6)	−0.0008 (6)	−0.0002 (6)
N29	0.0295 (7)	0.0310 (8)	0.0268 (8)	−0.0002 (6)	−0.0011 (6)	0.0006 (6)
C30	0.0215 (8)	0.0346 (9)	0.0291 (9)	0.0017 (7)	−0.0002 (7)	−0.0030 (7)
C31	0.0340 (9)	0.0383 (10)	0.0303 (9)	−0.0017 (8)	−0.0014 (8)	0.0003 (8)
C32	0.0363 (10)	0.0373 (10)	0.0424 (11)	−0.0042 (8)	−0.0012 (8)	−0.0028 (8)
C33	0.0253 (9)	0.0460 (11)	0.0396 (11)	−0.0003 (8)	−0.0028 (8)	−0.0137 (8)
Cl34	0.0433 (3)	0.0645 (4)	0.0607 (4)	−0.0052 (3)	−0.0112 (3)	−0.0283 (3)
C35	0.0346 (10)	0.0545 (12)	0.0308 (10)	0.0021 (9)	−0.0076 (8)	−0.0049 (9)
C36	0.0354 (10)	0.0397 (10)	0.0307 (10)	0.0005 (8)	−0.0051 (8)	0.0023 (8)

### Geometric parameters (Å, °)

**Table:**

F1—C4	1.332 (2)	F19—C22	1.331 (2)
F2—C4	1.331 (2)	F20—C22	1.333 (2)
F3—C4	1.335 (2)	F21—C22	1.333 (2)
C4—C5	1.553 (3)	C22—C23	1.553 (3)
C5—O6	1.213 (2)	C23—O24	1.210 (2)
C5—C7	1.453 (2)	C23—C25	1.453 (3)
C7—N10	1.322 (2)	C25—N28	1.324 (2)
C7—C9	1.459 (2)	C25—C27	1.455 (2)
O8—C9	1.217 (2)	O26—C27	1.219 (2)
C9—H9	0.9500	C27—H27	0.9500
N10—N11	1.2924 (19)	N28—N29	1.289 (2)
N11—C12	1.414 (2)	N29—C30	1.418 (2)
N11—H11	0.8800	N29—H29	0.8800
C12—C18	1.382 (2)	C30—C36	1.378 (3)
C12—C13	1.388 (2)	C30—C31	1.388 (3)
C13—C14	1.384 (2)	C31—C32	1.378 (3)
C13—H13	0.9500	C31—H31	0.9500
C14—C15	1.376 (3)	C32—C33	1.383 (3)
C14—H14	0.9500	C32—H32	0.9500
C15—C17	1.380 (3)	C33—C35	1.374 (3)
C15—Cl16	1.7444 (17)	C33—Cl34	1.7418 (19)
C17—C18	1.384 (2)	C35—C36	1.388 (3)
C17—H17	0.9500	C35—H35	0.9500
C18—H18	0.9500	C36—H36	0.9500
			
F2—C4—F1	106.75 (15)	F19—C22—F20	106.75 (15)
F2—C4—F3	107.72 (16)	F19—C22—F21	107.22 (15)
F1—C4—F3	107.31 (15)	F20—C22—F21	107.53 (16)
F2—C4—C5	111.72 (15)	F19—C22—C23	110.29 (16)
F1—C4—C5	110.14 (16)	F20—C22—C23	112.13 (15)
F3—C4—C5	112.91 (15)	F21—C22—C23	112.62 (15)
O6—C5—C7	124.78 (17)	O24—C23—C25	124.99 (18)
O6—C5—C4	117.46 (17)	O24—C23—C22	117.30 (17)
C7—C5—C4	117.76 (15)	C25—C23—C22	117.70 (16)
N10—C7—C5	114.86 (15)	N28—C25—C23	115.36 (16)
N10—C7—C9	125.69 (16)	N28—C25—C27	125.67 (17)
C5—C7—C9	119.46 (15)	C23—C25—C27	118.95 (16)
O8—C9—C7	122.43 (16)	O26—C27—C25	122.08 (18)
O8—C9—H9	118.8	O26—C27—H27	119.0
C7—C9—H9	118.8	C25—C27—H27	119.0
N11—N10—C7	121.04 (14)	N29—N28—C25	121.73 (15)
N10—N11—C12	119.13 (14)	N28—N29—C30	118.97 (14)
N10—N11—H11	120.4	N28—N29—H29	120.5
C12—N11—H11	120.4	C30—N29—H29	120.5
C18—C12—C13	121.04 (15)	C36—C30—C31	120.69 (17)
C18—C12—N11	117.95 (15)	C36—C30—N29	118.84 (16)
C13—C12—N11	121.01 (15)	C31—C30—N29	120.47 (16)
C14—C13—C12	119.29 (16)	C32—C31—C30	119.47 (18)
C14—C13—H13	120.4	C32—C31—H31	120.3
C12—C13—H13	120.4	C30—C31—H31	120.3
C15—C14—C13	119.29 (16)	C31—C32—C33	119.50 (18)
C15—C14—H14	120.4	C31—C32—H32	120.2
C13—C14—H14	120.4	C33—C32—H32	120.2
C14—C15—C17	121.74 (16)	C35—C33—C32	121.38 (18)
C14—C15—Cl16	119.69 (14)	C35—C33—Cl34	119.53 (16)
C17—C15—Cl16	118.57 (14)	C32—C33—Cl34	119.09 (16)
C15—C17—C18	119.14 (16)	C33—C35—C36	119.10 (18)
C15—C17—H17	120.4	C33—C35—H35	120.4
C18—C17—H17	120.4	C36—C35—H35	120.4
C12—C18—C17	119.49 (16)	C30—C36—C35	119.85 (18)
C12—C18—H18	120.3	C30—C36—H36	120.1
C17—C18—H18	120.3	C35—C36—H36	120.1
			
F2—C4—C5—O6	117.94 (19)	F19—C22—C23—O24	−0.9 (2)
F1—C4—C5—O6	−0.5 (2)	F20—C22—C23—O24	−119.74 (19)
F3—C4—C5—O6	−120.46 (19)	F21—C22—C23—O24	118.8 (2)
F2—C4—C5—C7	−61.9 (2)	F19—C22—C23—C25	178.66 (15)
F1—C4—C5—C7	179.66 (15)	F20—C22—C23—C25	59.8 (2)
F3—C4—C5—C7	59.7 (2)	F21—C22—C23—C25	−61.6 (2)
O6—C5—C7—N10	−177.83 (17)	O24—C23—C25—N28	−177.47 (18)
C4—C5—C7—N10	2.0 (2)	C22—C23—C25—N28	3.0 (2)
O6—C5—C7—C9	1.8 (3)	O24—C23—C25—C27	4.1 (3)
C4—C5—C7—C9	−178.39 (15)	C22—C23—C25—C27	−175.47 (16)
N10—C7—C9—O8	−2.0 (3)	N28—C25—C27—O26	0.5 (3)
C5—C7—C9—O8	178.45 (16)	C23—C25—C27—O26	178.74 (17)
C5—C7—N10—N11	−179.81 (14)	C23—C25—N28—N29	179.78 (15)
C9—C7—N10—N11	0.6 (2)	C27—C25—N28—N29	−1.9 (3)
C7—N10—N11—C12	−179.37 (14)	C25—N28—N29—C30	−179.92 (14)
N10—N11—C12—C18	174.08 (14)	N28—N29—C30—C36	−174.13 (15)
N10—N11—C12—C13	−6.2 (2)	N28—N29—C30—C31	6.3 (2)
C18—C12—C13—C14	0.2 (2)	C36—C30—C31—C32	0.5 (3)
N11—C12—C13—C14	−179.47 (15)	N29—C30—C31—C32	−179.94 (16)
C12—C13—C14—C15	−0.9 (2)	C30—C31—C32—C33	−0.2 (3)
C13—C14—C15—C17	1.0 (3)	C31—C32—C33—C35	−0.3 (3)
C13—C14—C15—Cl16	−179.66 (13)	C31—C32—C33—Cl34	179.43 (14)
C14—C15—C17—C18	−0.3 (3)	C32—C33—C35—C36	0.5 (3)
Cl16—C15—C17—C18	−179.69 (13)	Cl34—C33—C35—C36	−179.23 (14)
C13—C12—C18—C17	0.4 (2)	C31—C30—C36—C35	−0.3 (3)
N11—C12—C18—C17	−179.87 (14)	N29—C30—C36—C35	−179.87 (15)
C15—C17—C18—C12	−0.4 (2)	C33—C35—C36—C30	−0.2 (3)

### Hydrogen-bond geometry (Å, °)

**Table:**

*D*—H···*A*	*D*—H	H···*A*	*D*···*A*	*D*—H···*A*
N11—H11···O8	0.88	2.01	2.6746 (18)	131
N29—H29···O26	0.88	2.03	2.679 (2)	130
N29—H29···O26^i^	0.88	2.42	3.2159 (19)	150
C27—H27···O6^ii^	0.95	2.59	3.491 (2)	158
C36—H36···O26^i^	0.95	2.52	3.323 (3)	143

Symmetry codes: (i) −*x*, −*y*, −*z*+2; (ii) *x*−1, *y*, *z*.
